# SET domain-containing Protein 4 (SETD4) is a Newly Identified Cytosolic and Nuclear Lysine Methyltransferase involved in Breast Cancer Cell Proliferation

**Published:** 2013-01-21

**Authors:** Jerusa Araújo Quintão Arantes Faria, Natássia Caroline Resende Corrêa, Carolina de Andrade, Ana Carolina de Angelis Campos, Rubens dos Santos Samuel de Almeida, Thiago Souza Rodrigues, Alfredo Miranda de Goes, Dawidson Assis Gomes, Fábio Pittella Silva

**Affiliations:** 1 Department of Biochemistry and Immunology, Universidade Federal de Minas Gerais (UFMG), Belo Horizonte, MG, Brazil; 2 Laboratory of Molecular Pathology of cancer, Faculty of Health Sciences, University of Brasília (UnB), Brasília, DF, Brazil; 3 Computer department, Federal Center of Technological Education of Minas Gerais, Belo Horizonte, MG, Brazil

## Abstract

Cancer is comprised of a multitude of epigenetic abnormalities, including the global loss and regional gain of DNA methylation as well as alterations in histone methylation. Here, we characterize a new methyltransferase, SET domain-containing protein 4 (SETD4), which is involved in breast carcinogenesis. Quantitative real-time PCR (qPCR) showed elevated expression levels of SETD4 in several breast cancer cell lines. SETD4 overexpression was confirmed by western blot analysis suggesting a correlation between high expression of SETD4 and a lack of the estrogen receptor (ER) in breast cancer. In addition, cell fractionation studies and confocal immunofluorescence revealed the nuclear and non-nuclear localization of this new protein. SETD4 knockdown in breast cancer cell lines significantly suppressed their proliferation and delayed the G1/S cell cycle transition without affecting apoptosis. Furthermore, western blot analysis showed that knockdown of SETD4 decreased cyclin D1 expression, revealing the involvement of SETD4 in cell cycle regulation. These data imply that SETD4 plays a crucial role in breast carcinogenesis and could be a novel molecular target for the development of new strategies for the diagnosis and treatment of breast cancer.

## Introduction

Breast cancer is the most frequently diagnosed cancer and the leading cause of cancer death in women worldwide. An estimated 1.38 million women globally were diagnosed with breast cancer in 2008, accounting for nearly one-quarter of all cancers diagnosed in women [1]. Cancer can evolve from a combination of epigenetic and genetic abnormalities, resulting in deregulated gene expression and function [2].

Histone modification regulates chromatin structure as well as transcriptional activation and repression and includes acetylation, ribosylation, ubiquitylation, phosphorylation, sumoylation, and methylation. Histone modification occurs at selected residues that function in a combined or sequential fashion to dictate ‘histone codes’ that are closely linked to biological outcomes [3]. In breast cancer, abnormal histone modification in combination with DNA hypermethylation is frequently associated with the epigenetic silencing of tumor suppressor genes and genomic instability. Understanding the mechanisms of deregulation of histone tail post-translational modifications and their contribution to breast tumorigenesis is critically important for developing novel targeted therapies for breast cancer patients [4].

Histone lysine methylation has been shown to be catalyzed exclusively by the conserved SET domain family proteins. The exception to this rule is the DOT1 family, members of which are structurally unrelated to the SET domain proteins [5,6]. Recent data have revealed that SET domain-containing proteins can catalyze the lysine methylation of non-histone cellular proteins, such as p53, VEGFR, ERα and NF-κB [7].

Several lysine methyltransferases have shown altered expression in many diseases, including cancer [8]. For instance, EZH2 and SMYD3 are overexpressed in various types of cancer, including breast cancer and have been closely linked to breast carcinogenesis through distinct mechanisms [9,10]. In the present paper, we report the characterization of SETD4, a human SET domain-containing protein that is overexpressed in human ER-negative breast cancer cells. Furthermore, SETD4 up-regulation is also associated with the proliferation of breast cancer cell line MDA-MB 231. Our data should yield new insights into breast carcinogenesis and could contribute to the development of novel approaches for breast cancer treatment.

## Materials and Methods

### Bioinformatics tools and comparative modeling

Analysis of functional domains was performed using the NCBI Conserved Domains and Pfam databases [11,12]. The 3D molecular models of SETD4 were built by comparative modeling. Human SET domain-containing protein 3 (SETD3) from the PDB database (3SMT) was used as a template for modeling. Molecular models were generated using Modeller (version 9.7) [13], considering the presence of heteroatoms (SAM, *S*-adenosyl methionine) and loop refinement. One hundred candidate models were generated for each protein system, and each model was evaluated using stereochemical quality Ramachandran plots generated using Procheck (version 3.5.4) [14] and energy values according to Prosa (ProSa 2003) [15]. Visualization and manipulation of molecular images were performed using Pymol (version 1.2) [16].

### Cell culture

HCC-1954-BL, HCC-1954, CAMA-1, SKBR-3, MCF-7, MDA-MB 231, MDA-MB 436 and MDA-MB 468 human cell lines were purchased from the American Type Cell Culture Collection (ATCC). Two primary breast cancer cell lines were used: MACL-1 and MGSO-3. These cell lines were established from fragments of breast tumors at our laboratory [17]. Primary cultures of normal human mammary epithelial cells were isolated from reduction mammoplasties, as previously described [18]. Cells were grown in Dulbecco’s modified Eagle’s medium (DMEM; Sigma-Aldrich, St. Louis, MO, USA) supplemented with 10% fetal bovine serum (Cripion Biotechnology, Andradina, SP, Brazil) and penicillin/streptomycin (100 U/mL; Life Technologies, Carlsbad, CA, USA) at 37°C and 5% CO_2_. MDA-MB 231 cells were transfected with shSETD4 (TG301750; OriGene, Rockville, MD, USA) using Lipofectamine 2000 (Life Technologies). Stable clones were selected using 800 μg/ml G418 (Sigma-Aldrich) for 14 days.

### Tumor specimens

Sample collection was approved by the Research Ethics Committee of the Faculty of Health Sciences, University of Brasília, Brazil, based on resolution 196/96 of the National Heath Council/Brazilian Ministry of Health, project number 025/09. Written informed consent was obtained from all participants. The samples were collected from surgically removed breast tissue from patients with breast cancer immediately after surgery at the University of Brasilia Hospital. The clinical and histopathological characteristics of the patients are summarized in Table 1.

### cDNA synthesis and qPCR analysis

Total RNA was extracted using TRIzol (Life Technologies) treated with RNase-free DNase (Promega, Madison, WI, USA) and the synthesis of cDNA was performed with RevertAid^™^ H Minus M-MuLV RT (Fermentas, Waltham, MA, USA) using an oligo(dT) adapter primer. Gene expression was quantified using the comparative Ct (2^−ΔΔCt^) method. RPS27a and β-actin were used as endogenous control genes for data normalization.

### cDNA microarray

Double-strand cDNA from MACL-1, MGSO-3 and normal cells was synthesized using SuperScript Double-Stranded cDNA Synthesis Kit (Life Technologies) and Human Gene Expression 12×135K array chip (Roche NimbleGen Inc., Madison WI, USA) was used. Data were normalized on software NimbleScan (Roche), as described by Bolstad and co-workers [19]. Gene information was generated using RMA algorithm [20]. Volcano plots were generated and only genes that displayed more than a twofold-change and p<0.05 on Student’s t test were considered as differentially expressed [21].

### Sample preparation and immunoblotting

Subcellular fractionation and immunoblots were performed as described previously [22,23]. Commercially available antibodies against SETD4 (Santa Cruz Biotechnology, Santa Cruz, CA, USA), lamin B1 (Abcam, Cambridge, MA, USA), α-tubulin (Sigma-Aldrich) and cyclins D1, D2 and D3 (Cell Signaling Technology, Beverly, MA, USA) were used. Blots were visualized by enhanced chemiluminescence and quantitatively analyzed using Image software.

### Immunofluorescence

Confocal immunofluorescence was performed as described [23]. The samples were incubated with a 1:200 dilution of rabbit polyclonal anti-human SETD4 (Santa Cruz Biotechnology). The nuclei were counterstained with 0.2 μg/mL Hoechst (Life Technologies). All confocal images were collected with a Zeiss LSM 510 or Zeiss 5 Live confocal microscope using a 63x, 1.4 NA objective lens with excitation at 488 nm and observation at 505–550 nm to detect Alexa Fluor 488 staining and excitation at 405 nm and observation at 420–460 nm to detect Hoechst staining.

### MTT assay

3-(4,5-Dimethylthiazol-2-yl)-2,5-diphenyltetrazolium bromide (MTT; Life Technologies) was used to determine cell viability [24]. After 72 h, cells were plated, 210 μL of medium and 170 μL MTT at 5 mg/mL were added to each well. The blue formazan crystals were dissolved in 210 μL of a 10% SDS/HCl (Sigma-Aldrich) solution and the absorbance was read at 595 nm in a microplate reader (BioTek Instruments, Inc., Winooski, VT, USA). The results are expressed as a percentage of the absorbance present in treated cells compared with control cells.

### Clonogenic assay

Cell survival was measured using the colony formation assay. Briefly, 900 cells were seeded in 35-mm plates and incubated for 10 days, after which colonies were stained with a mixture of 6.0% glutaraldehyde (Sigma-Aldrich) and 0.5% crystal violet (Sigma-Aldrich) and then rinsed with water. Surviving fractions were normalized against the plating efficiency of non-transfected cells.

### Cell cycle analysis

A total of 1×10^5^ cells were lysed with 300 μL of a hypotonic solution containing 0.5% Triton X-100 (Sigma-Aldrich) and 50 μg/mL propidium iodide (Life Technologies). The cells were incubated at 4°C for 2 h and analyzed in a Guava Easycyte 6L flow cytometer (Millipore, Billerica, MA, USA). Data were analyzed using FlowJo software (version 7.2.5).

### Statistical analysis

Data are expressed as the mean ± Standard Deviation (SD) and compared using Tukey’s test among groups after one-way ANOVA.

## Results

### The structure of human SETD4 is related to that of human SETD3

**Figure 1:**
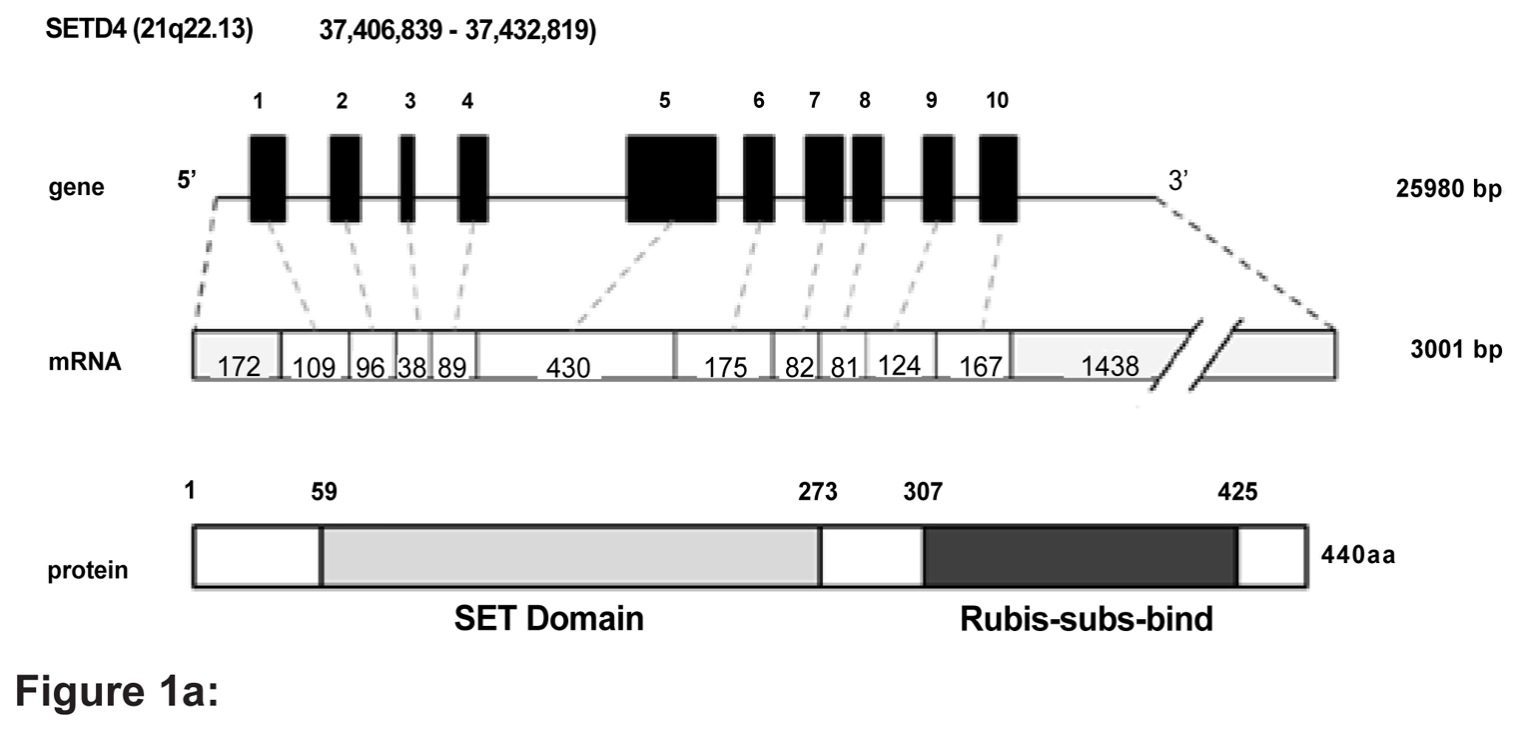

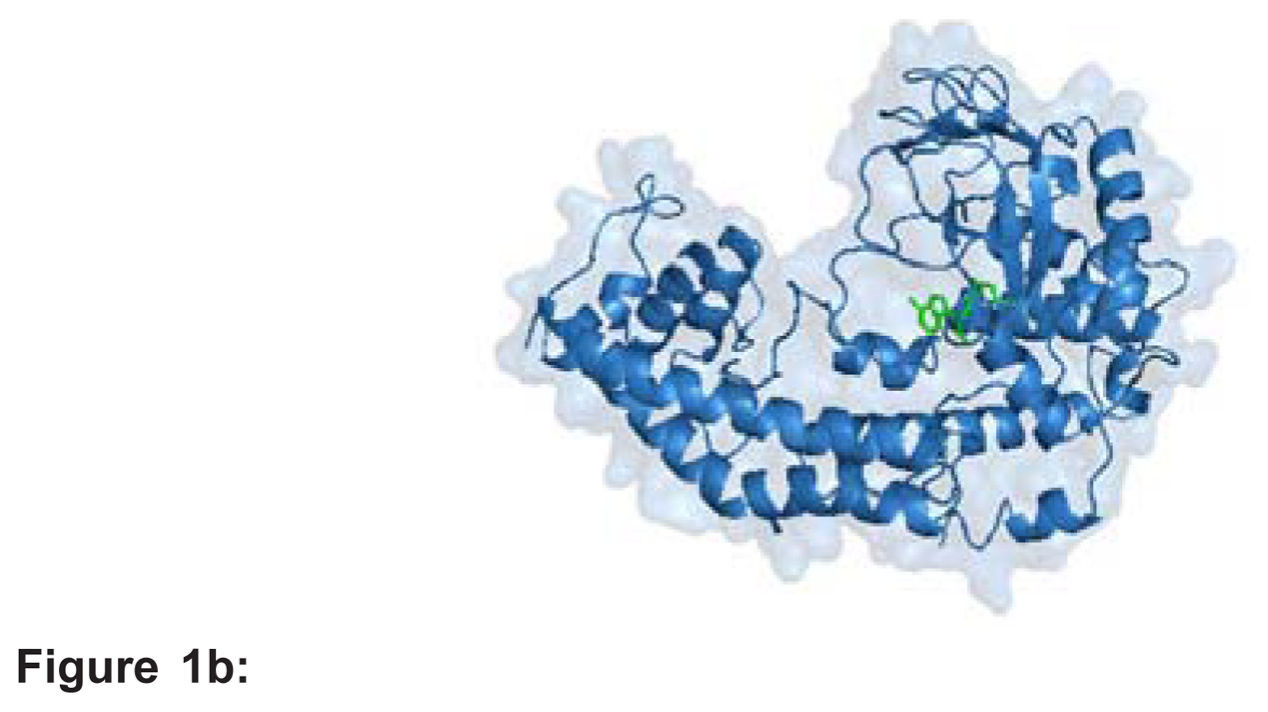

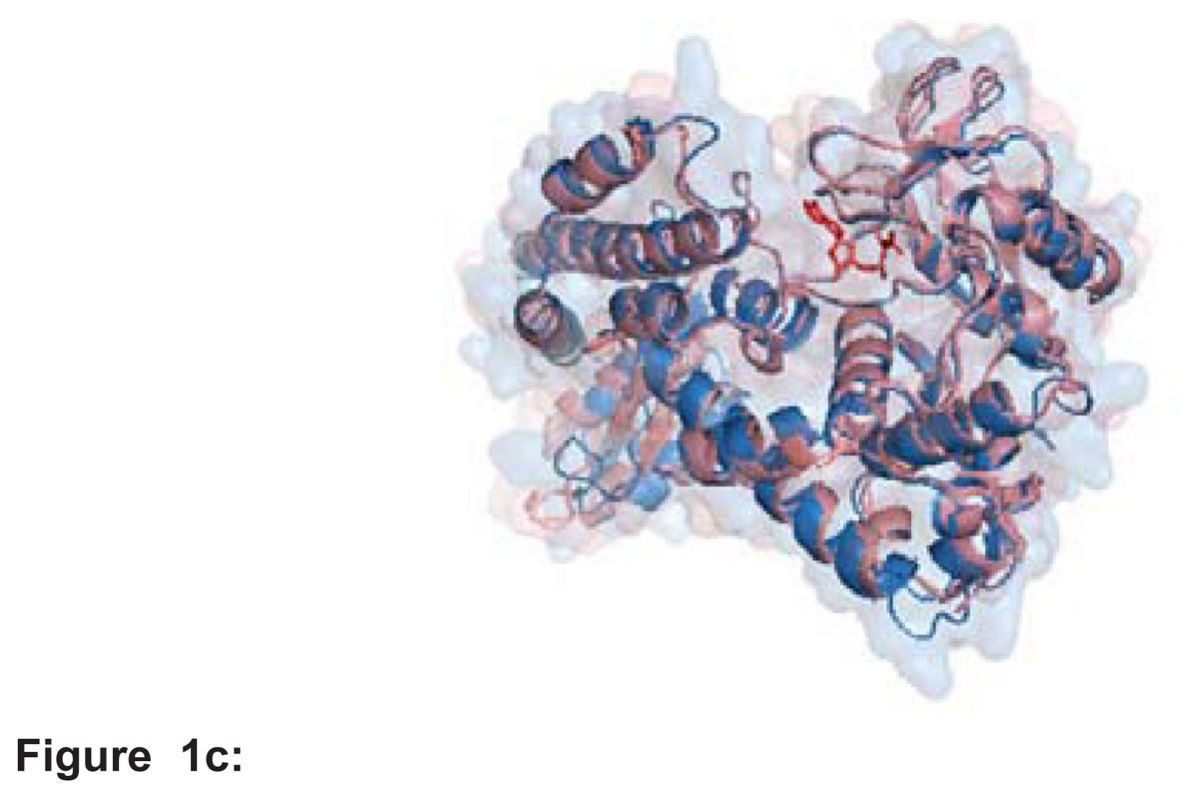

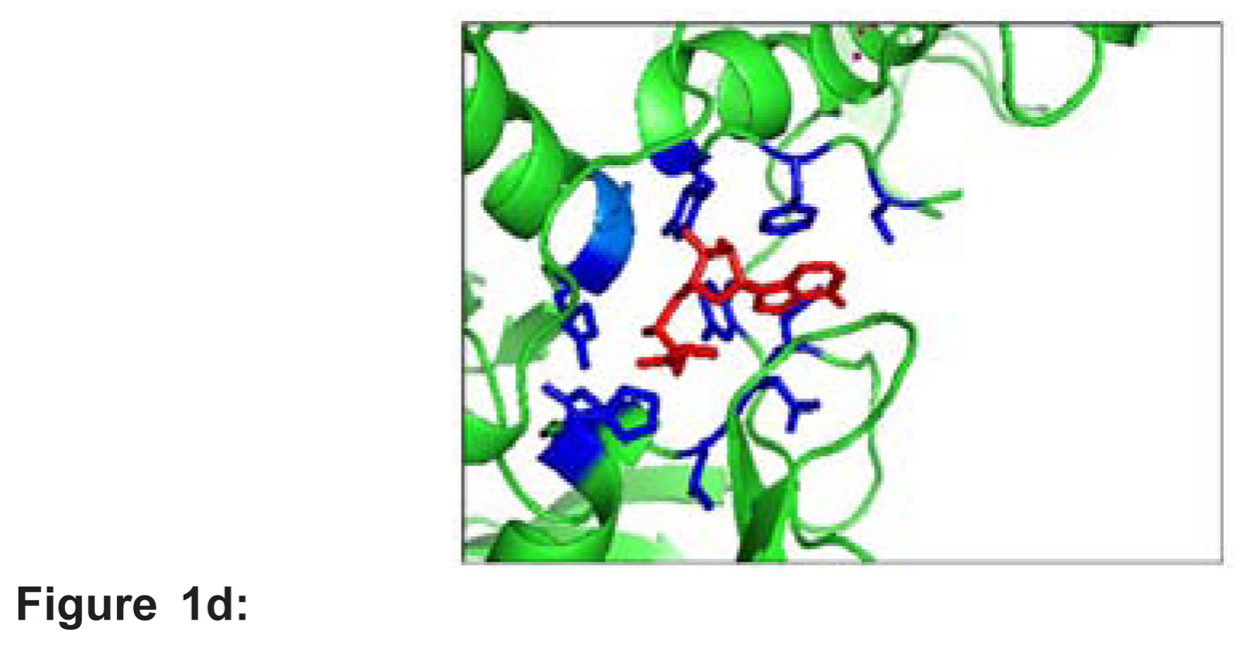
**Figure 1a:** Domain structure of SETD4 and comparative modeling. Schematic representation of the gene and primary protein structures of SETD4. Black boxes in the genes and white boxes in the mRNAs denote exons. The numbers above each gene are exon numbers. The numbers within the exons indicate their sizes in nucleotides. Thin lines in the genes indicate the introns and untranslated regions of the first and the last exons (mRNA, gray boxes). Gray and black boxes indicate SET and Rubis-subs-bind domains, respectively. **Figure 1b:** 3D structure of the SETD4 protein showed the presence of the cofactor SAM (green) inside the structure. This model was deposited in the Protein Model Data Base (PMDB). **Figure 1c:** Superposition of template and model structures reveals high similarity between SETD3 and SETD4. SAM is shown in red. **Figure 1d:** Detailed view of the region of SETD4 that interacts with SAM. Conserved amino acid residues are shown in blue, and SAM is shown in red.

We predict that SETD4 is located at 21q22.13, which transcribes an mRNA of 3001 bp, corresponding to a 44-kDa protein containing 440 amino acids. The encoded protein has a SET domain at its N-terminus (59–273) and a Rubis-subs-bind domain at its C-terminus (307–425) (Figure 1A). The SET domain is a characteristic motif of enzymes that catalyze the addition of methyl groups to specific lysine residues in histone or non-histone proteins [5,8]. The Rubis-subs-bind domain, also referred to as the Rubisco LSMT substrate-binding domain, permits the binding of the protein to a substrate, such as the N-terminal tails of histones and other targets [25]. The three-dimensional structure of human SET domain-containing protein 3 (PDB ID: 3SMT chain A, at 2.04 Å resolution) was used as a template for homology modeling. Our human SETD4 model was deposited in the Protein Model DataBase (PMDB) with the identification number PM0078503 (http://mi.caspur.it/PMDB/) (Figure 1B). SETD4 exhibited high stereochemical quality (92.8% of residues in the allowed regions of the Ramachandran plot) and a high probability to represent a native-like conformation (ProSa Z-score 8.07). Despite the low identity of the residues (24%), the secondary structure profiles of both proteins were highly similar and the only structure discrepancy was found in the loop regions. The overlap of the template structure and our model are shown in Figure 1C.

The SET domain has a unique structural fold and differs from other classes of protein methyltransferases that also use the cofactor *S*-Adenosyl-L-Methionine (SAM) as the methyl donor cofactor. The proper positioning of SAM in the structure of SET proteins is critical to their catalysis [26]. Figure 1D shows 13 protein residues interacting with SAM, which are also conserved in our SETD4 model. The residues Asn236, His237 and Tyr272 (using SETD4 residue numbering) are invariant among the SET proteins and are in the correct position to play catalytic roles [26]. Consistent with the catalytic importance of these residues, mutation of any of these residues in SET proteins (in DIM-5 or SET7/9) dramatically reduces catalytic activity [26,27].

Overall, the molecular model obtained is highly informative because it includes alignment optimization, loop refinement, secondary structure predictions and SAM positioning.

### SETD4 expression is elevated in ER-negative breast cancer

**Figure 2:**
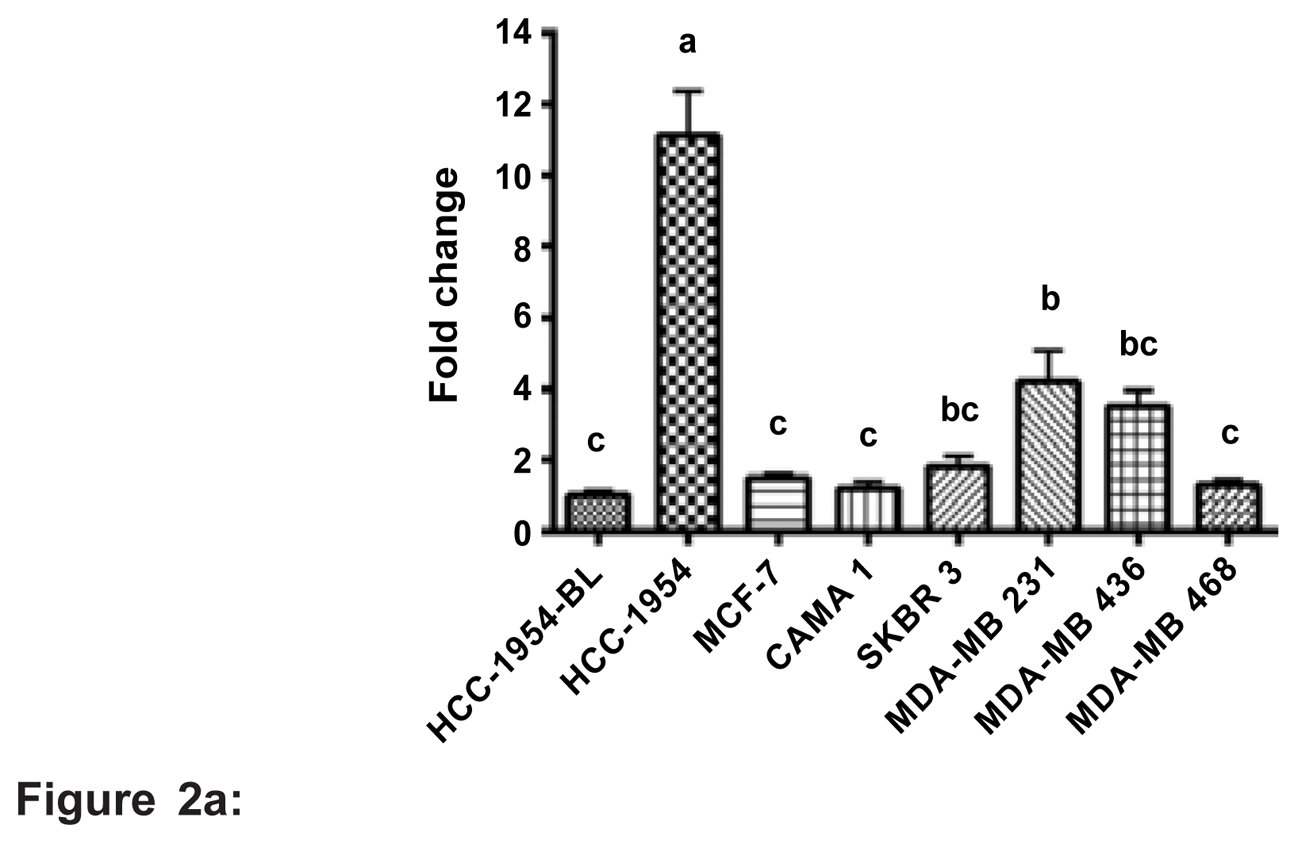

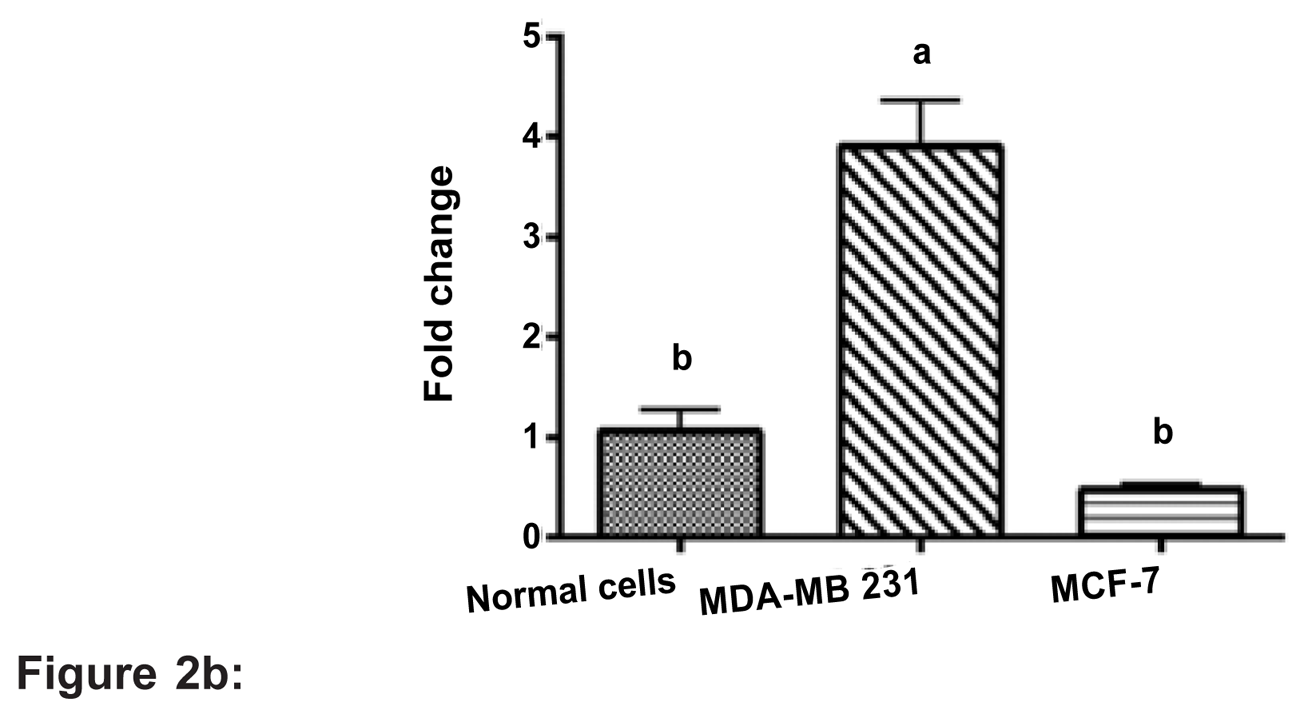

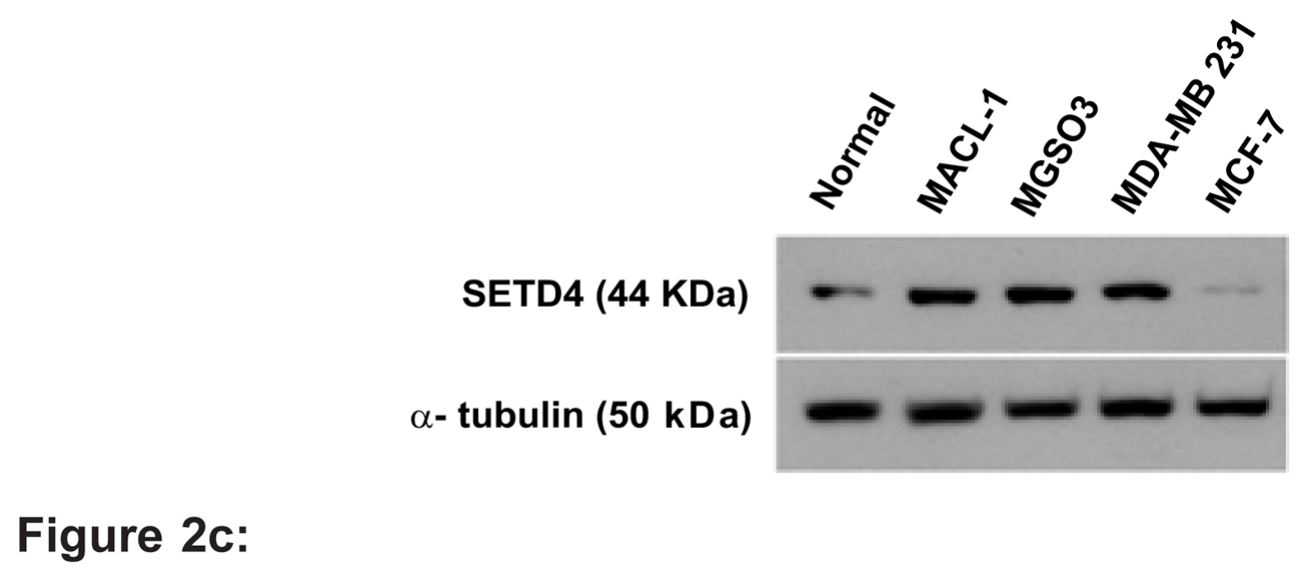

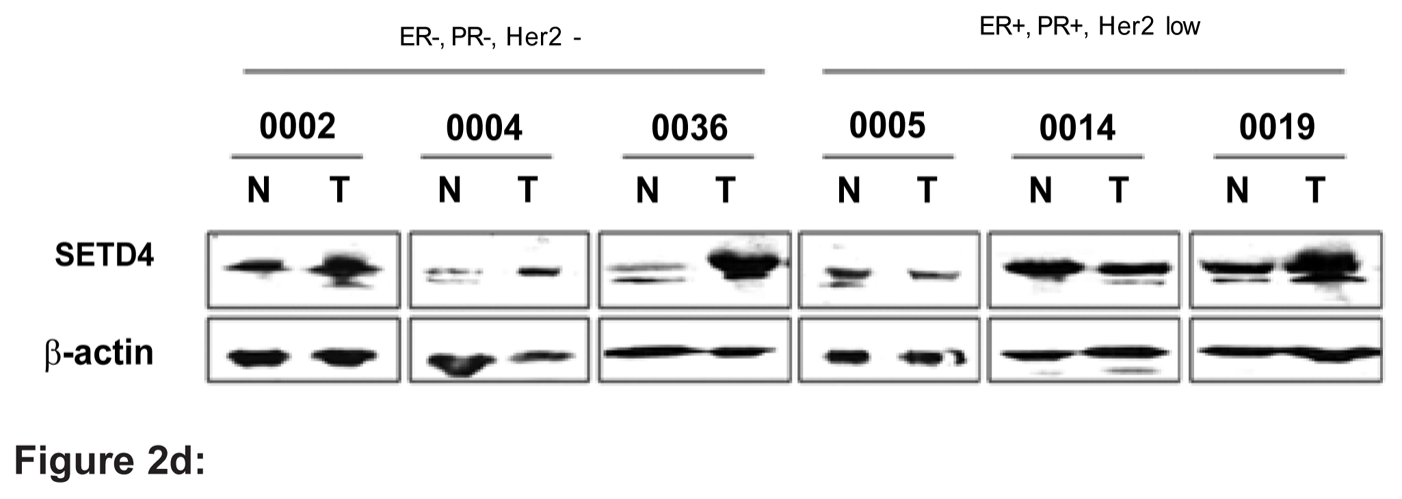

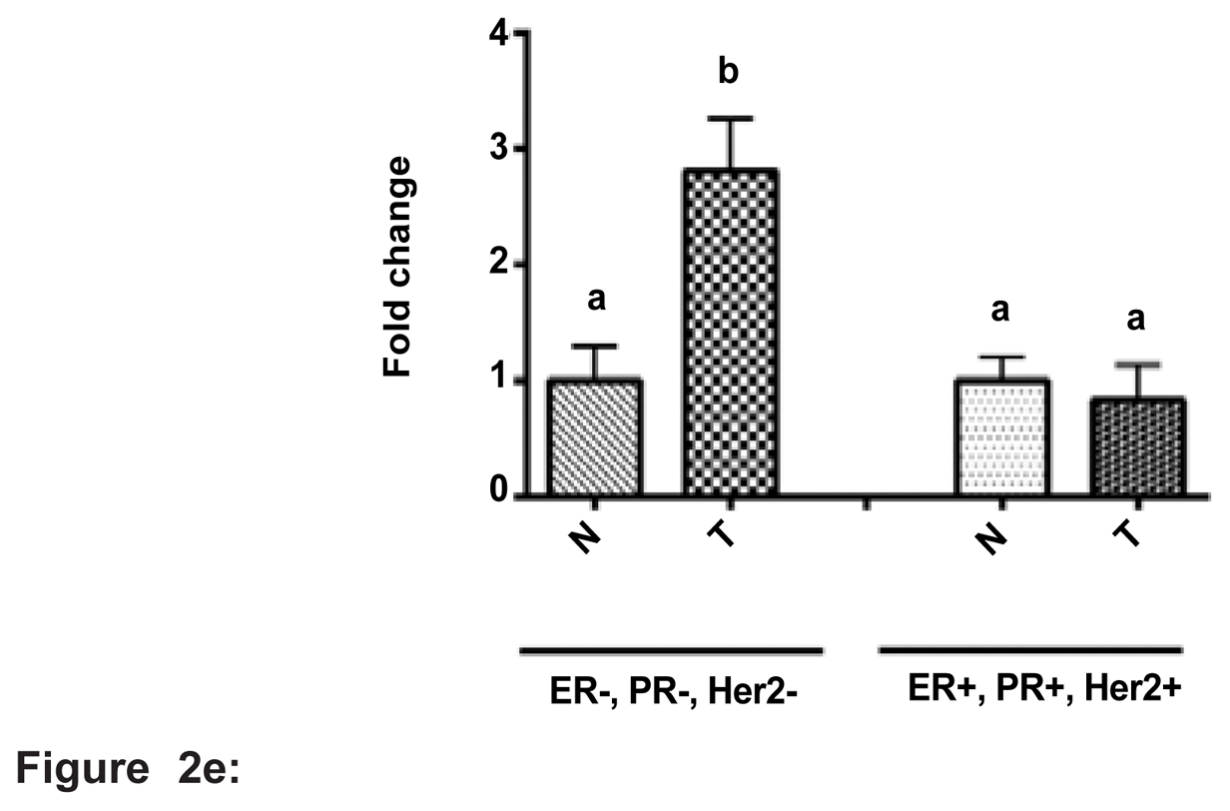
**Figure 2a:** SETD4 is overexpressed in ER-negative breast cancer. SETD4 mRNA expression was evaluated by qPCR. The SETD4 expression values for the HCC-1954, MCF-7, CAMA-1, SKBR-3, MDA-MB 231, MDA-MB 436 and MDA-MB 468 cells were normalized to that of the HCC-1954-BL control cells. **Figure 2b:** The expression of SETD4 in MDA-MB 231 and MCF-7 cells was normalized relative to normal, non-cancerous cells. β-actin and RPLS27a were used as endogenous controls. **Figure 2c:** Immunoblot analysis of total protein extracts of breast cancer cell lines. Densitometric analysis showed that SETD4 is up-regulated in MACL-1, MGSO-3, MDA-MB 231 cells (ER-negative) and down-regulated in MCF-7 cells (ER-positive). **Figure 2d:** Immunoblots were performed to quantify the expression of SETD4 in breast cancer tissues. **Figure 2e:** Densitometric analysis confirmed that triple-negative samples showed elevated expression of SETD4 compared with tumors that were positive for ER, PR and HER2. Means followed by the same letter indicate no significant difference by Tukey’s test (p<0.001).

Quantitative PCR analysis was used to examine SETD4 mRNA expression in a panel of cells, including the non-tumorigenic line HCC-1954-BL and several breast cancer cells. As shown in Figure 2A, the expression of SETD4 was elevated in the HCC-1954, SKBR-3, MDA-MB 231 and MDA-MB 436 cell lines compared with the control HCC-1954-BL cell line, a lymphoblast cell line isolated from the same individual from who the HCC-1954 breast cancer cells were isolated (p<0.001). A similar profile was observed when the relative expression of SETD4 was normalized to normal breast cells (Figure 2B). The MDA-MB 231 cells showed high levels of SETD4, while the MCF-7 cells did not show altered expression compared to the control (p<0.001). To confirm this data, SETD4 protein expression was evaluated in normal cells and breast cancer cells by western blot analysis (Figure 2C). The MACL-1, MGSO-3 and MDA-MB 231 cells revealed high expression of the SETD4 protein, whereas the MCF-7 cells showed reduced expression. The microarray analysis data for the MACL-1 and MGSO-3 cells showed that SETD4 is up-regulated in these primary cell lines compared to normal cells (non-cancerous) (Table 2). Cell lines classified as ER-positive have been observed to show reduced expression of SETD4 (MCF-7 and CAMA-1). In contrast, cell lines with high expression of this protein are ER-negative (HCC-1954, MDA-MB 231 and MDA-MB 436) [28] as well as the primary cell lines MACL-1 and MGSO-3 [29]. To assess this correlation, we detected SETD4 protein expression in breast cancer tissues. Total extracts from six paired breast cancer tissues (T) and their corresponding non-tumorous tissues (N) were used in western blotting analyses (Table 1). In Figure 2D, tumors that were classified based on receptor markers showed similar SETD4 expression profiles. Data normalization revealed high expression of SETD4 in triple-negative tumors and no change in samples positive for ER, PR and HER2 (p<0.001) (Figure 2E). Our preliminary data suggest a negative correlation between the overexpression of SETD4 and expression of the ER and more studies are necessary to validate this observation. Two different commercially available antibodies that we used to immunoblot SETD4 did not work for immunohistochemical staining.

### SETD4 is localized in the nucleus and cytosol

**Figure 3:**
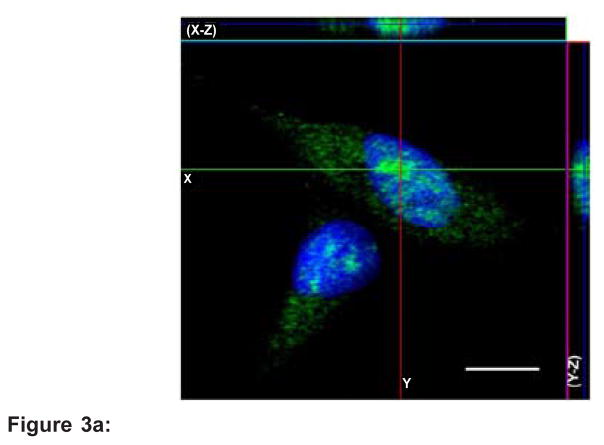

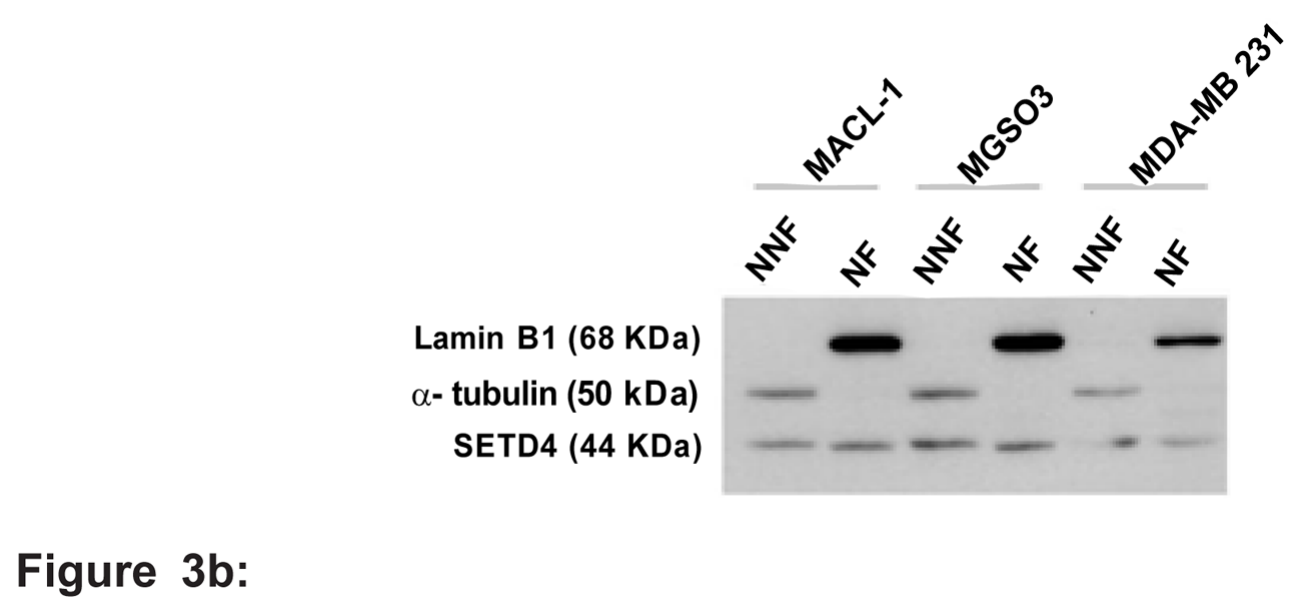
**Figure 3a:** SETD4 is localized in both the cytoplasm and nucleus. MDA-MB 231 cells were grown on glass slides prior to fixation and immunofluorescence microscopy using an antibody specific for the SETD4 protein and a secondary antibody conjugated to Alexa Fluor 488 (green). Nuclear staining with Hoechst is shown in blue. Serial optical sections were collected for three-dimensional reconstruction; x-z sections are shown at the top and y-z sections are shown on the right. Scale bar=10 μm. **Figure 3b:** Immunoblot analysis of extracts of the non-nuclear (NNF) and nuclear fractions (NF) of breast cancer cells (MACL-1, MGSO-3 and MDA-MB 231). α-Tubulin and lamin B1 were used as purity controls for the non-nuclear and nuclear fractions, respectively.

Confocal immunofluorescence was performed to directly visualize the subcellular localization of SETD4 in MDA-MB-231 cells. Serial optical sections were collected for three-dimensional reconstruction. We observed a fine, wide and meshwork-like staining pattern of SETD4 in the cytoplasm and a punctate pattern of SETD4 in the nucleus (Figure 3A). To confirm these data, cell fractionation was performed using MACL-1, MGSO-3 and MDA-MB 231 breast cancer cells, which have elevated SETD4 expression. Immunoblot analysis confirmed that SETD4 appears in both the nuclear and non-nuclear fractions (Figure 3B).

### SETD4 plays an essential role in the growth regulation of breast cancer cells

**Figure 4:**
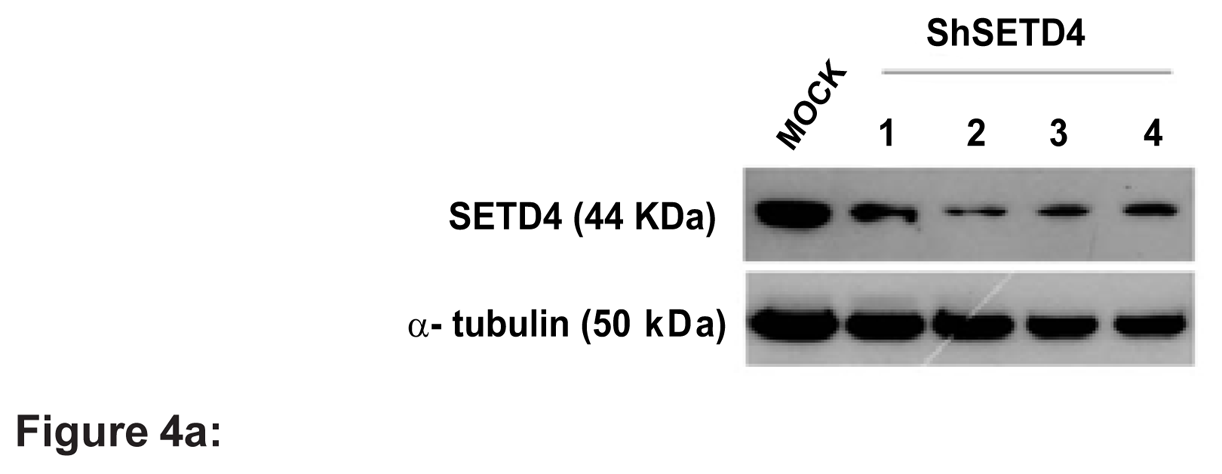

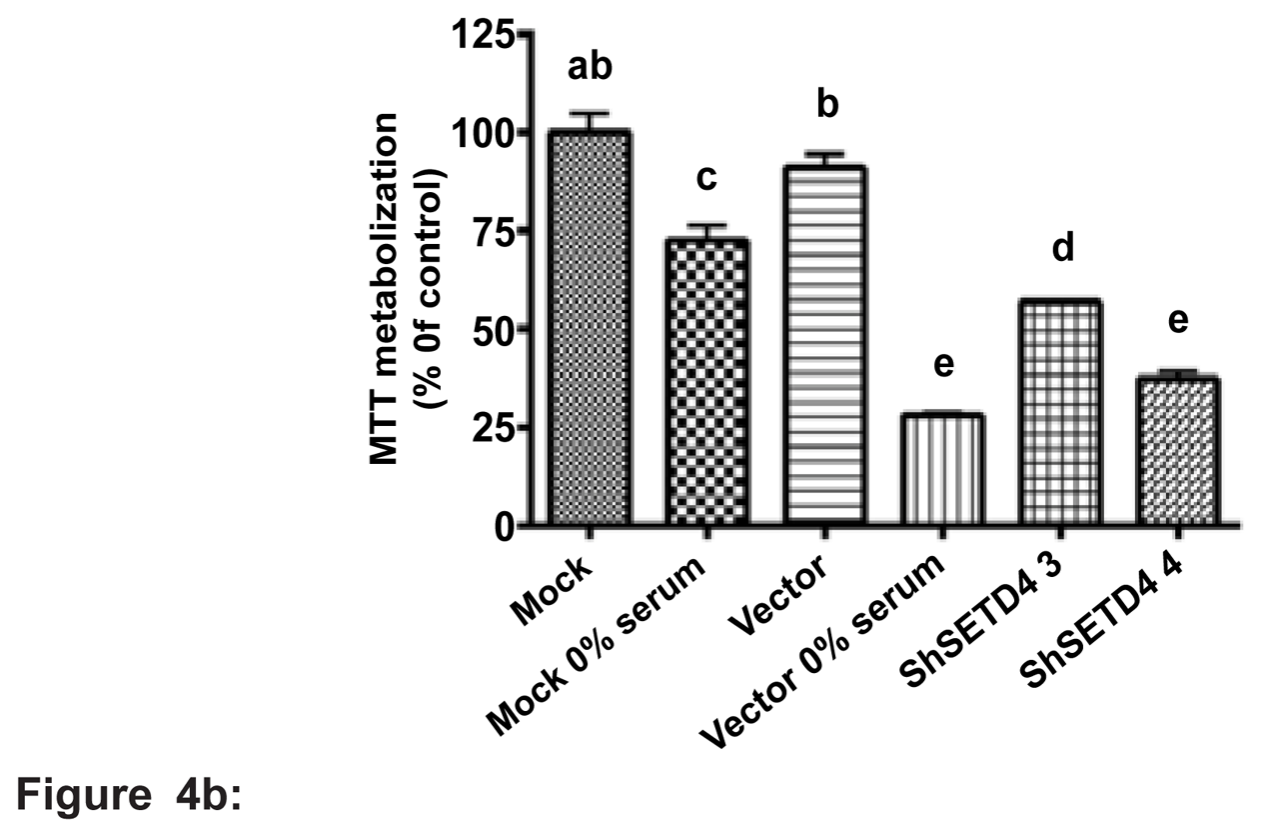

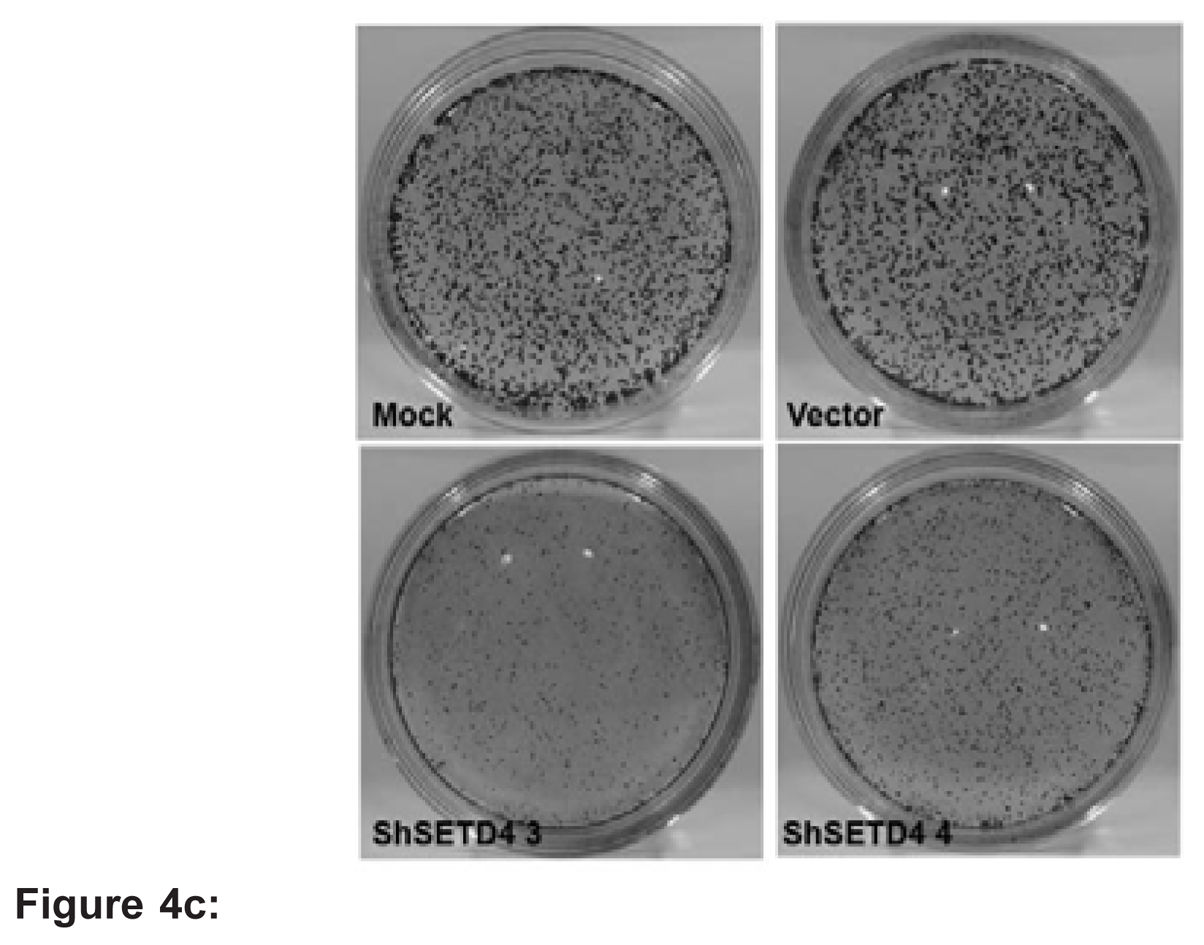

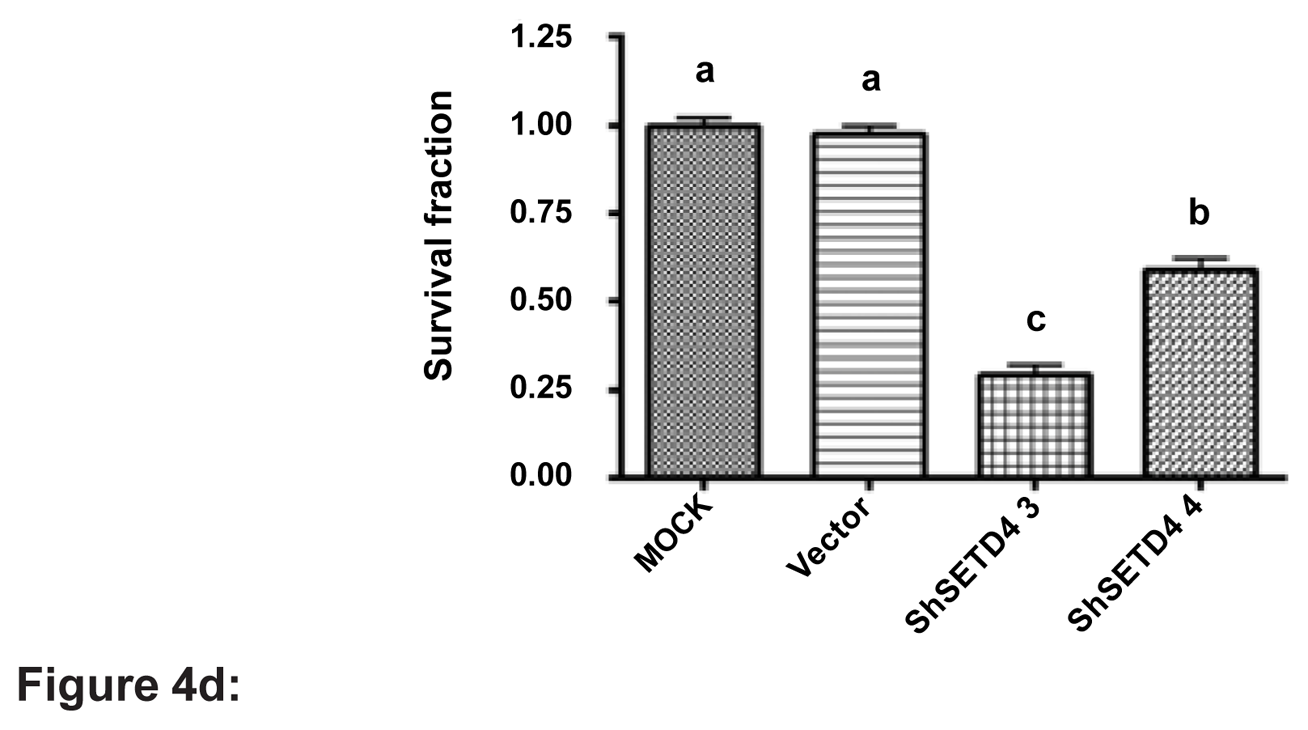

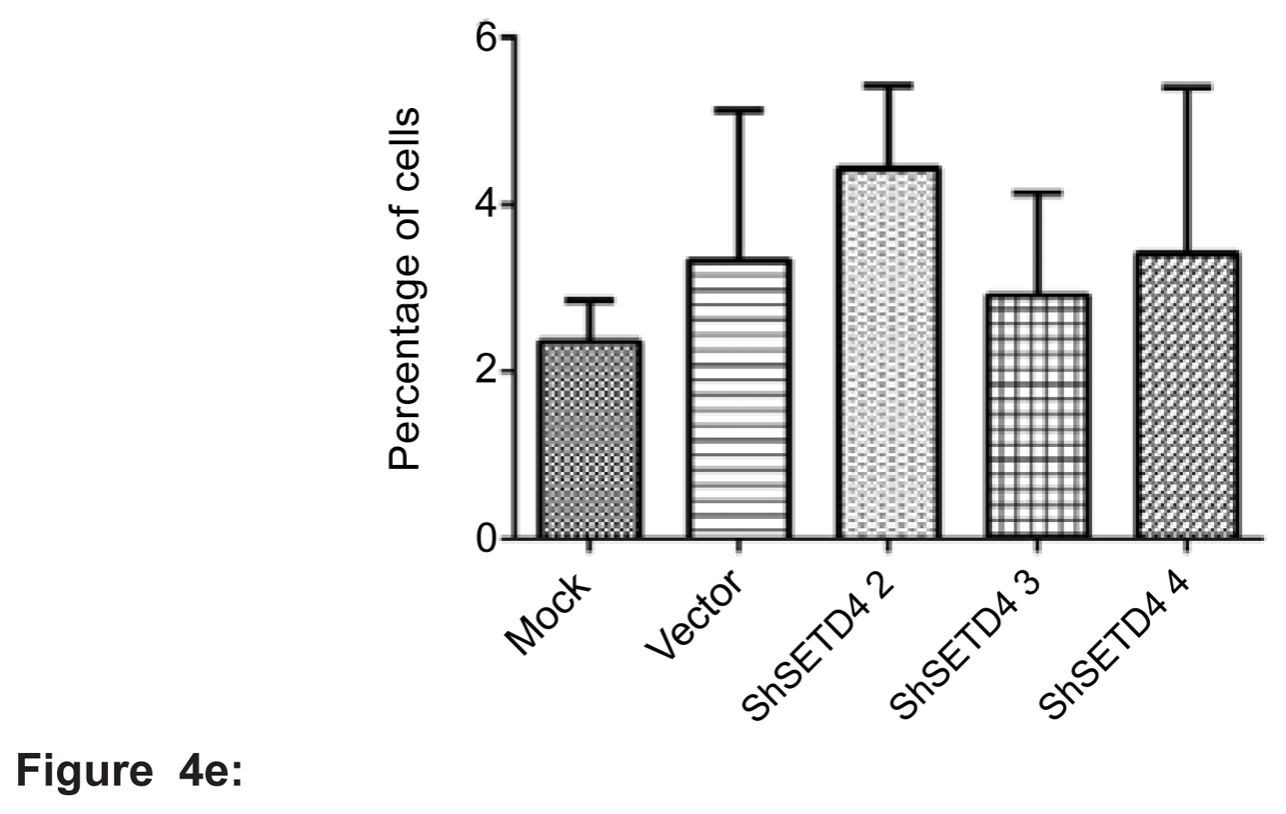

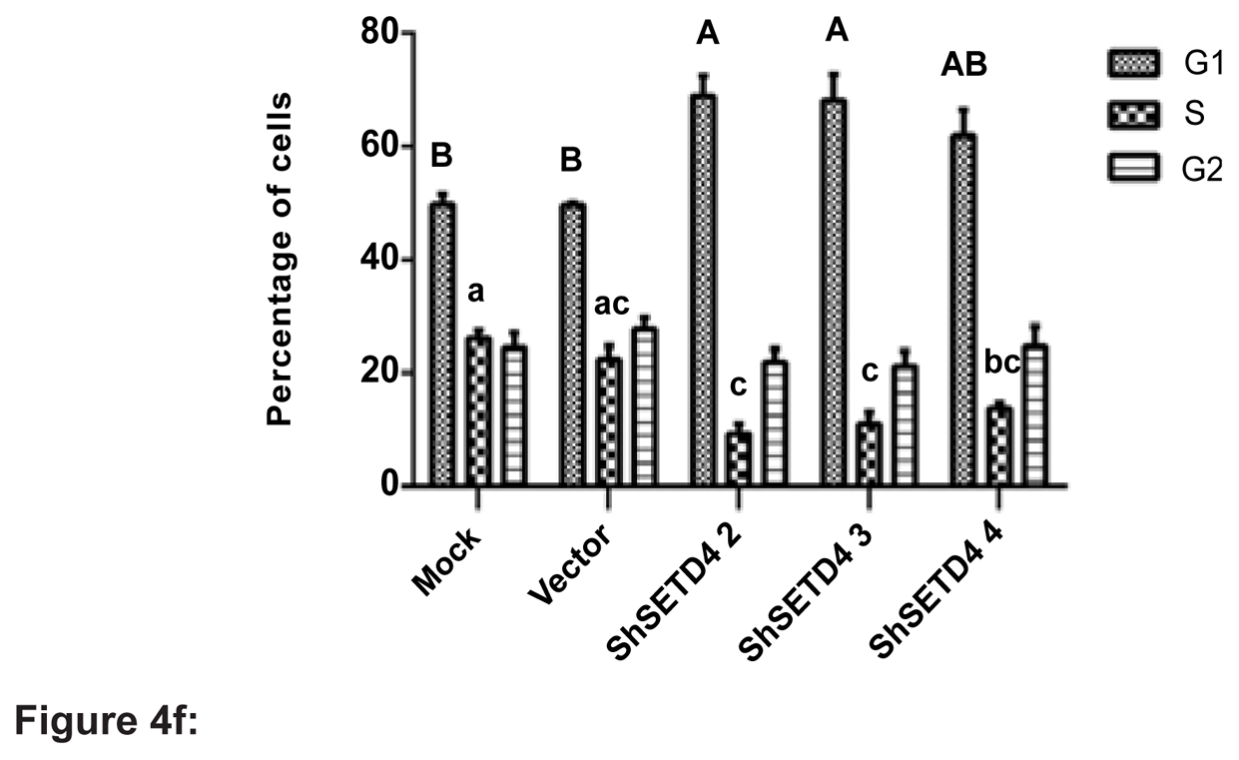

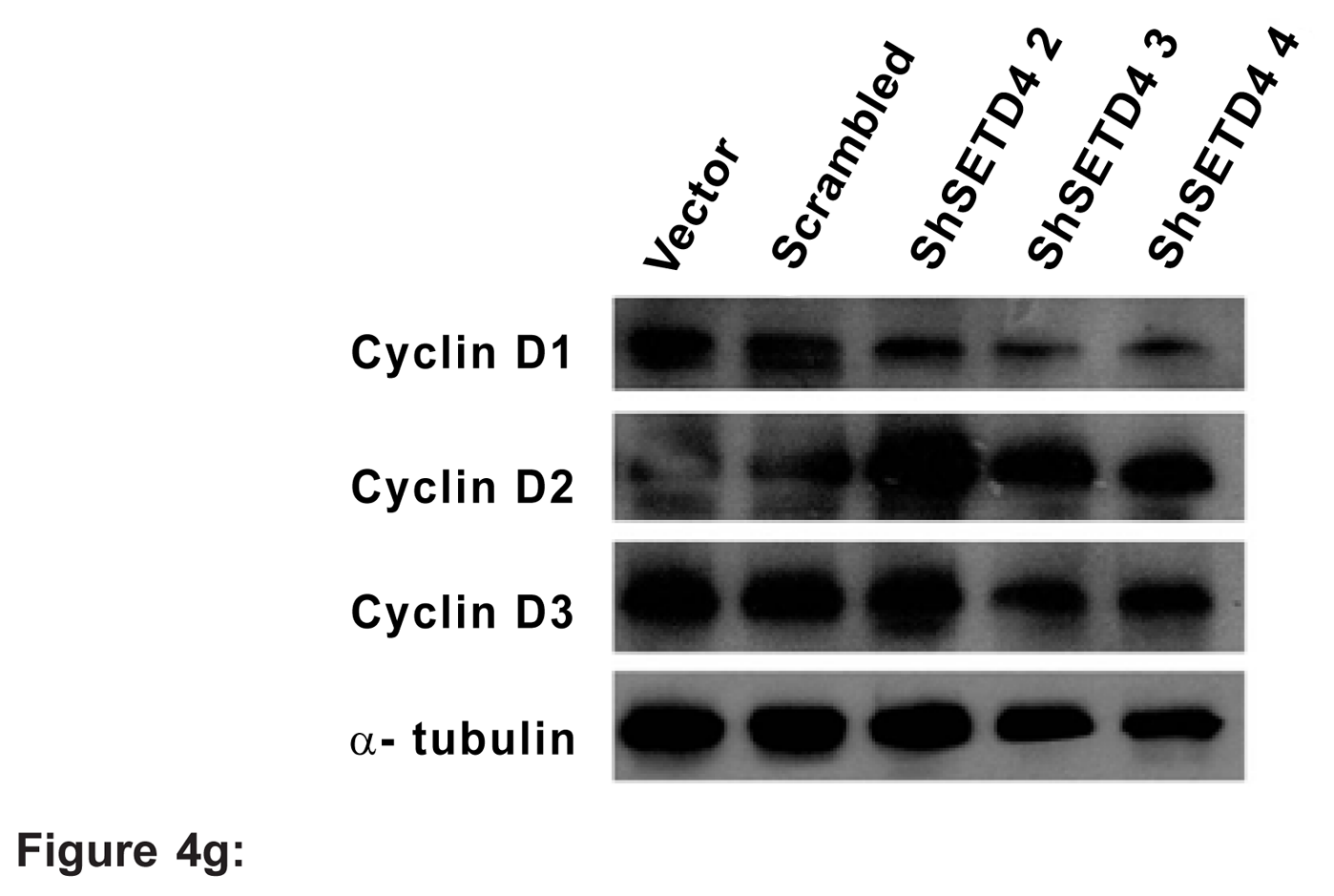

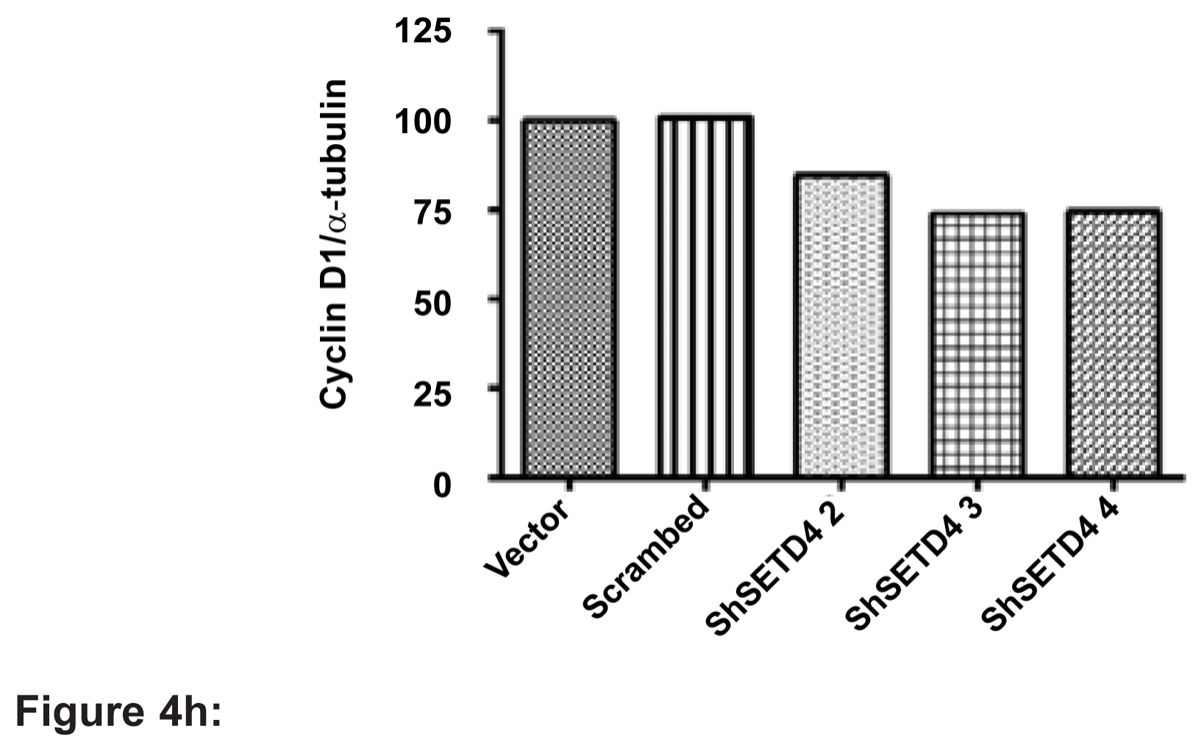
**Figure 4a:** Knockdown of SETD4 leads to the significant growth suppression of cancer cells. Lysates from the MDA-MB 231 stable knockdown cells (ShSETD4 1–4) were immunoblotted with antibodies against SETD4 and α-tubulin (as an internal control) to evaluate SETD4 knockdown. **Figure 4b:** Effects of SETD4 knockdown on the viability of MDA-MB 231 cells, as measured by the MTT assay. Mock (non-transfected MDA-MB 231 cells), vector (cells transfected with empty vector) and two independent clones (shSETD4 3 and 4) were plated in medium containing FBS for 72 h. Mock and vector cells were also maintained in medium without FBS (0% serum). The results are expressed relative to mock cells. Means followed by the same letter indicate no significant difference by Tukey’s test (p<0.001). The results are representative of three independent experiments. **Figure 4c:** Colony formation assay of shSETD4 cells. Cells were grown until colonies formed (10 days) and then stained, fixed and counted. **Figure 4d:** The mean survival fraction ± SEM of triplicate wells was normalized to mock cells based on the plating efficiency. Tukey’s test (p<0.001). **Figure 4e:** Flow cytometric analysis of sub-G1 DNA content revealed no significant differences between groups of cells by Tukey’s test (p<0.001). **Figure 4f:** Cell cycle distribution was analyzed by flow cytometry after staining with propidium iodide. Data from the numerical analysis in which cells were classified by cell cycle status are expressed as percentages. Means followed by the same letter indicate no significant difference by Tukey’s test (p<0.05). Uppercase and lowercase letters represent the analysis of G1 and S phase, respectively. The results are expressed as the percentage of events from a total of 20000 events (n=3). **Figure 4g:** Immunoblots were performed to quantify the expression of cyclin D1, D2 and D3. α-Tubulin was used as an internal control. **Figure 4h:** Densitometric analysis confirmed reduced cyclin D1 expression to approximately 30%.

To determine the significance of SETD4 in human carcinogenesis, we examined whether SETD4 is involved in the growth regulation of cancer cells. After confirming the elevated expression of SETD4 in breast tumors and cell lines, we knocked down SETD4 expression in MDA-MB 231 cells using short-hairpin constructs. To investigate whether the elevated expression of SETD4 plays a crucial role in the proliferation of breast cancer cells, we tested four different shRNA constructs specific for SETD4 (OriGene, Rockville, MD, USA). Clones were established using G418 selection (Figure 4A). SETD4 knockdown significantly suppressed MTT metabolism in breast cancer cells (Figure 4B). Interestingly, suppression of SETD4 drastically reduced the colony formation of MDA-MB-231 breast cancer cells (Figures 4C and 4D). The decrease in proliferation was not due to cell death, as shown by the determination of sub-G1 DNA content (Figure 4E). We next evaluated the detailed cell cycle status of SETD4 knockdown cells and confirmed that the proportion of cancer cells in S phase was significantly decreased (Figure 4F). Concomitantly, the percentage of cancer cells in G1 phase was increased, indicating that SETD4 could be a critical factor in the regulation of the G1-S transition in cancer cells. We next investigated the effect of SETD4 knockdown on the relative expression of cell cycle regulators, such as the G1 phase-specific cyclin D. The SETD4 knockdown cells showed reduced cyclin D1 expression compared with cells transformed with vector. Conversely, the reduction in SETD4 did not affect the expression of cyclins D2 and D3 (Figures 4G and 4H).

## Discussion

The human methyltransferasome includes 208 known and putative members. To date, 30% of these proteins have been linked to disease states, of which 22 SET proteins have been associated with cancer or other diseases in humans or mouse models [8,30]. The present study provides the first characterization of a novel lysine methyltransferase, SETD4, which is related to cancer. Given that there is no X-ray crystal or NMR structures available for SETD4; we developed a protein model by comparative modeling based on the SETD3 structure to understand the structure and function of the SETD4 protein. SETD3, SETD4 and SETD6 are grouped into methyltransferase class VII, which represents classical non-histone SET domain methyltransferases; proteins of this class are most similar to the plant Rubisco methyltransferase [30]. The comparative analysis of the 3D molecular models of SETD4 and SETD3 supports the hypothesis of SETD4 as a functional lysine methyltransferase. However, its specific substrates and modification sites remain to be disclosed.

The cellular fractionation and immunolocalization of SETD4 indicated a wide distribution of the protein in the cytosol and a punctual localization in the nucleus (Figure 3). The regulatory role of protein methylation is not restricted to the histone code but is also linked to several other cellular processes [7], as this modification occurs in both cytosolic and nuclear proteins. EZH2 protein expression was observed primarily in the nucleus, and its expression was significantly increased in invasive carcinoma and breast cancer metastases [31]. EZH2 in the cytoplasm is involved in the regulation of receptor-induced actin polymerization, indicating a role for lysine methylation in the cytoplasm [32]. SMYD3 has a histone H3K4-specific methyltransferase activity and can methylate the VEGF receptor when localized in the cytosol [9,33]. Thus, like these methyltransferases, it is possible that SETD4 acts on different targets and in different cellular compartments.

Similar to our results for SETD4 (Figure 2), EZH2 transcript and protein expression are elevated in breast cancer [31]. EZH2 overexpression occurs mainly in basal-type tumors, which are characterized by a ER-, PR-, and Her-2/neu-negative status as well as low levels of the BRCA1 protein. The down-regulation of EZH2 in aggressive ER-negative breast cancer cells greatly decreases their proliferative capacity and rate of progression through the cell cycle [34].

A major novel finding presented in our study is that SETD4 down-regulation in aggressive ER-negative breast cancer cells greatly decreases their proliferative capacity and rate of progression through the cell cycle. The number of breast cancer samples from patients used in this study was limited and further work will be necessary to validate this observation. Furthermore, we found that SETD4 knockdown caused an arrest at the G1/S transition in the cell cycle via a reduction in the cyclin D1 level. EZH2 knockdown prolongs the doubling time of ER-negative breast cancer cell lines and causes an arrest at the G2/M transition of the cell cycle, with corresponding changes in mitotic Cdc25C, Cdc2 and Cdc2-Tyr15 phosphorylation [34]. In addition, SMYD2 has been shown to play a crucial role in the G1/S transition through the methylation of the RB1 protein, resulting in augmented E2F transcriptional activity and a promotion of cell cycle progression [35]. Although the exact mechanism regarding how SETD4 affects the level of cyclin D1 remains unclear, its involvement in cell cycle regulation adds new insights to breast carcinogenesis.

The correlation between various methyltransferases and breast cancer highlights the importance of this protein family in the progression of this disease. Further work will reinforce the importance of SETD4 as a target for breast cancer therapy and will help elucidate the mechanisms involved in its activity. Our findings reveal the importance of SETD4 in breast carcinogenesis and may contribute to the identification of novel strategies to treat ER-negative breast tumors.

## Figures and Tables

**Table 1:** Clinical and histopathological characteristics of breast cancer samples. The identification of tumor tissue from the removed sample was based on the histopathological examination, and all types of breast cancer were included. Samples were selected based on tumor content (minimally 80% tumor), as determined by microscopic pathological analysis. A sample of normal tissue was collected from each patient whenever possible. All cases represent ductal carcinoma *in situ*.

ID	Sample Malignancy	Breast	Age	Histological grade	Stage	Chemotherapy	c-erbB2	ER	PR
002	80%	Left	64	Intermediate	YPT4PN1M0	Yes	−	−	−
004	100%	Left	58	High	T3N2aM0	Yes	−	−	−
036	80%	Left	56	High	T2N1aM0	Yes	−	−	−
005	80%	Right	60	Low	T2N1 M0	No	low	+	+
014	100%	Right	61	High	T2N0 M0	No	low	+	+
019	80%	Left	54	Intermediate	T4dN3 M0	Yes	low	+	+

c-erbB2: HER2/neu Epidermal Growth Factor Receptor; ER: Estrogen Receptor; PR: Progesterone Receptor.

**Table 2:** Gene expression level (fold change) of SETD4 in the MACL-1 and MGSO-3 cell lines compared to normal cells (non-cancerous), as assessed by cDNA microarray. RNA samples from MACL-1, MGSO-3 and normal cells were extracted using RNeasy Mini Kit (Qiagen, Venlo, The Netherlands), following manufacturer’s protocol and cleaned from DNAse with RQ1 RNase-free DNAse (Promega). Double-strand cDNA was synthesized using SuperScript Double-Stranded cDNA Synthesis Kit (Life Technologies), according to manufacturer’s directions and cDNA quality was then evaluated on BioAnalyzer 2100 (Agilent Technologies, Palo Alto, CA, USA). Human Gene Expression 12×135K array chip (Roche NimbleGen Inc., Madison WI, USA) was used to assess gene expression profile with each cell line represented in triplicate. After Cy3 labeling, the cDNA was hybridized to the chip and slides were scanned at 532nm. Internal fluorescence was adjusted and sample tracking control was verified to exclude cross contamination between samples.

Cell line	Fold change	p-value
MACL-1	9.5	4.01×10^−6^
MGSO-3	3.43	0.025
